# Fecal Short-Chain Fatty Acids in Colorectal Cancer Patients Versus Healthy Controls: A Systematic Review and Meta-Analysis

**DOI:** 10.3390/jcm14248949

**Published:** 2025-12-18

**Authors:** Tomasz Sylwestrzak, Michalina Ciosek, Krzysztof Pastuszak, Tomasz Jastrzębski

**Affiliations:** 1 Department of Gynecology and Obstetrics, Medical University of Gdańsk, M. Skłodowskiej-Curie 3a Street, 80-210 Gdańsk, Poland; 2 Department of Surgical Oncology, Transplant Surgery and General Surgery, Medical University of Gdańsk, M. Skłodowskiej-Curie 3a Street, 80-210 Gdańsk, Poland; m.ciosek@gumed.edu.pl; 3 Laboratory of Translational Oncology, Intercollegiate Faculty of Biotechnology of the University of Gdańsk and the Medical University of Gdańsk, M. Skłodowskiej-Curie 3a Street, 80-210 Gdańsk, Poland; 4 Department of Algorithms and Systems Modelling, Faculty of Electronics, Telecommunications and Informatics, G. Narutowcza 11/12 Street, Gdańsk University of Technology, 80-233 Gdańsk, Poland; 5 Centre of Biostatistics and Bioinformatics, Medical University of Gdańsk, M. Skłodowskiej-Curie 3a Street, 80-210 Gdańsk, Poland; 6 Oncological Surgery Department, PCZ Brzeziny Hospital, M. Skłodowskiej-Curie 6 Street, 96-060 Brzeziny, Poland

**Keywords:** colon cancer, colorectal cancer, SCFA, butyrate, acetate, propionate, biomarker

## Abstract

**Background**: Short-chain fatty acids (SCFAs), the main microbial fermentation products in the colon, have immunometabolic and anti-neoplastic properties. Alterations in fecal SCFA profiles have been proposed as potential non-invasive biomarkers for colorectal cancer (CRC), but previous findings remain inconsistent. This systematic review and meta-analysis aimed to determine whether fecal acetate, propionate, and butyrate concentrations differ between patients with CRC and healthy individuals. **Methods**: A comprehensive search of PubMed, Web of Science and Cochrane Library was conducted on 18 September 2025. Eligible studies were observational, included adults with histologically confirmed CRC and healthy controls, and reported fecal concentrations of at least one SCFA quantified using validated analytical methods. Two independent reviewers performed study screening, data extraction, and risk-of-bias assessment. Random-effects models were applied to calculate pooled standardized mean differences (SMDs) with 95% confidence intervals (CIs). **Results**: Thirteen studies met inclusion criteria for qualitative synthesis, and four (141 CRC cases, 98 controls) were eligible for meta-analysis. Compared with healthy controls, patients with CRC had significantly lower fecal acetate (pooled SMD −0.37; 95% CI −0.63 to −0.10; *p* = 0.006; I^2^ = 0%) and butyrate (pooled SMD −0.59; 95% CI −1.10 to −0.07; *p* = 0.026; I^2^ = 64.4%), whereas propionate did not differ significantly (pooled SMD −0.02; 95% CI −0.85 to 0.82; *p* = 0.971; I^2^ = 89%). **Conclusions**: CRC is associated with reduced fecal butyrate and, to a lesser extent, acetate, suggesting impaired microbial fermentation. Propionate shows no consistent difference. SCFA profiling currently lacks sufficient standardization and validation for clinical application. Future harmonized, longitudinal studies integrating diet, microbiome, and metabolomic data are warranted to confirm SCFAs as reproducible biomarkers of CRC.

## Simple Summary

The gut microbiota produces short-chain fatty acids through the fermentation of dietary fiber, which play essential roles in maintaining intestinal health and regulating cellular metabolism. Alterations in these metabolites have been implicated in colorectal cancer development. This study systematically reviewed and analyzed published evidence comparing fecal concentrations of acetate, propionate, and butyrate between patients with colorectal cancer and healthy individuals. The findings indicate that colorectal cancer is associated with reduced levels of butyrate and acetate, whereas propionate remains largely unchanged. These results suggest a disruption of microbial fermentation processes within the colorectal environment. Although short-chain fatty acids show potential as non-invasive indicators of colorectal cancer-related metabolic changes, current evidence is limited by methodological variability. Standardized, large-scale studies are required to validate their diagnostic and prognostic value.

## 1. Introduction

Colorectal cancer (CRC) remains a major global health burden, ranking among the top three most common cancers and leading causes of cancer mortality [1]. Despite the availability of screening tools, there is a clear need for improved non-invasive biomarkers to enable earlier detection and risk stratification of CRC [2]. Notably, fewer than 5% of CRC cases are attributable to high-risk inherited syndromes, underscoring the significant contribution of environmental and dietary factors in most patients [3]. Diet-induced alterations in the gut microbiome and its metabolites have emerged as important modulators of colorectal carcinogenesis [4]. Fecal microbial metabolites are attractive biomarker candidates, and short-chain fatty acids (SCFAs), the main products of colonic fiber fermentation, are of particular interest; however, no SCFA measure is yet validated for clinical CRC screening or risk stratification, evidence is heterogeneous, and higher-quality prospective studies are needed [5,6,7].

SCFAs, primarily acetate, propionate, and butyrate, are abundant in the healthy colon and stool. They typically occur at an approximate molar ratio of 60:20:20 (acetate/propionate/butyrate) in the colonic lumen, reflecting the dominance of acetate-producing bacteria and the somewhat lower proportions of propionate and butyrate producers [8,9,10]. Total fecal SCFA concentrations in healthy individuals typically range from 50 to 150 mmol/L, although exact levels and acetate-to-propionate-to-butyrate ratios vary with diet, microbiota composition, and host factors [11]. SCFA, particularly butyrate, serve as a primary energy source for colonocytes and contribute to the maintenance of intestinal barrier integrity by enhancing tight-junction formation [12]. SCFAs modulate host physiology through cell-signaling pathways: they activate G-protein-coupled receptors (GPR41, GPR43, GPR109A) on immune and epithelial cells, influencing inflammatory responses, and inhibit histone deacetylases (HDACs), thereby regulating gene expression [13,14,15]. In patients with colorectal cancer, gut dysbiosis is frequently characterized by a loss of SCFA-producing bacteria and a consequent reduction in fecal SCFA concentrations [16]. In fact, CRC is associated with significant reductions in SCFA levels, and it is emphasized that SCFA normally act as tumor-suppressive, immunomodulatory metabolites in the colon [17]. In healthy tissue, SCFA help maintain T-cell homeostasis, inhibit inflammation, and downregulate oncogenic pathways [18,19,20]. Thus, depletion of SCFA levels may accompany colorectal tumorigenesis. However, empirical studies comparing fecal SCFA in CRC versus controls have yielded mixed results, with some finding lower SCFA levels in cancer patients while others report no statistically significant difference [21,22,23,24]. These discrepancies underscore the need for a systematic synthesis of the evidence.

Accordingly, we performed a systematic review and meta-analysis of observational studies measuring fecal SCFA concentrations in adults with confirmed CRC and healthy controls. While pooled SCFAs were previously shown to be lower in CRC [6], this study examines whether individual SCFA show consistent differences. We focused on the three main SCFA: acetate, propionate, butyrate; measured by validated analytical methods [25,26]. Our aim was to determine whether fecal SCFA levels are consistently altered in CRC, which could shed more light on gut microbiome–cancer interactions and the potential of SCFA as noninvasive biomarkers of colorectal cancer. This systematic review and meta-analysis is conducted in accordance with the Preferred Reporting Items for Systematic Reviews and Meta-Analyses (PRISMA) 2020 guidelines [27] (Supplementary Table S1). The protocol for this systematic review and meta-analysis was registered with PROSPERO CRD420251176944.

## 2. Methods

**Literature Search, Eligibility Criteria, and Screening:** We systematically searched PubMed, Web of Science, and Cochrane Library databases as well as additional sources on 18 September 2025, using a search strategy developed collaboratively by the lead reviewer and an experienced research librarian. Additional sources included reference list screening and citation tracking. Only English-language, full-text articles were considered. Title and abstract screening was independently conducted by two reviewers using Rayyan, a citation classification application [28]. Conflicts were resolved by discussion. Full-text screening of potentially eligible studies was performed by the same reviewers, with discrepancies resolved by consensus.

Eligible studies were required to have an observational design and to report fecal SCFA concentrations in adults (≥18 years) with histologically confirmed colorectal cancer, include a healthy control group, apply validated SCFA quantification techniques—gas chromatography (GC), gas chromatography–mass spectrometry (GC–MS), gas chromatography–time-of-flight mass spectrometry (GC–TOF-MS), high-performance liquid chromatography (HPLC), ultra-performance liquid chromatography–mass spectrometry (UPLC–MS), or proton nuclear magnetic resonance (^1^H-NMR)—and provide absolute or relative concentration data for at least one individual SCFA (acetate, propionate, or butyrate). Studies assessing other biological matrices (e.g., serum or urine), involving pediatric populations, or lacking quantitative SCFA data were excluded, as were review articles, conference abstracts, and case reports. Data extraction was performed independently by two reviewers using a standardized template, capturing study design, geographic location, year of publication, sample size, participant characteristics, analytical methods, units of measurement, and reported SCFA outcomes, with discrepancies resolved by consensus. Only studies that fulfilled the eligibility criteria for the systematic review, provided extractable numerical SCFA data, and demonstrated an acceptable risk-of-bias profile were subsequently included in the quantitative meta-analysis.

**Data Extraction:** Two reviewers independently extracted data using a standardized Microsoft Excel form. Extracted information included: author, publication year, country, study design, inclusion and exclusion criteria, sample timing, dietary or antibiotic exclusions, sample storage conditions, SCFA quantification method and units, group sizes, mean and standard deviation (SD) (or median and interquartile range (IQR) or confidence interval (CI)) for acetate, propionate, and butyrate (CRC and control groups), reported covariates and conflicts of interest or funding sources.

**Figure 1:**
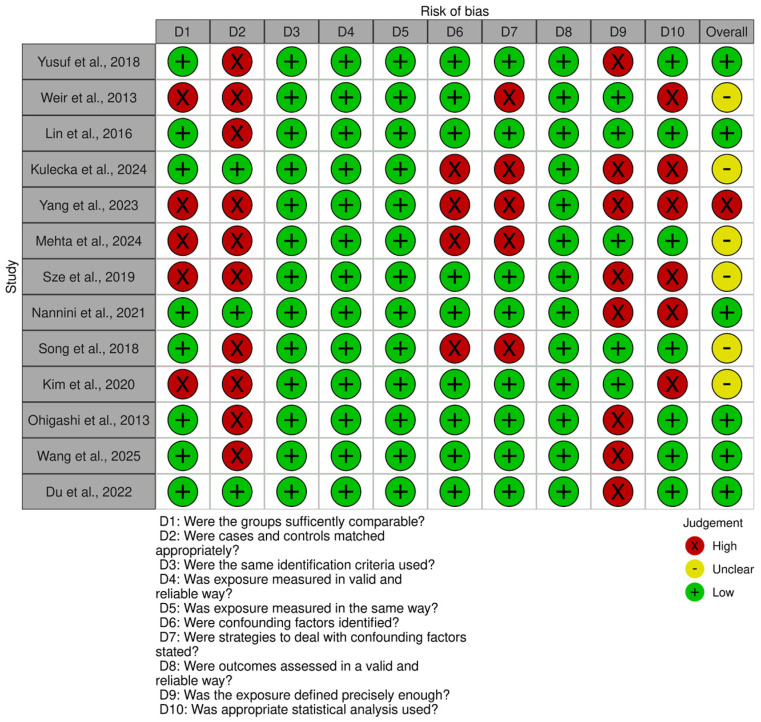
Risk-of-bias traffic light plot. Visual summary of individual study judgments across ten domains of bias assessment using the Joanna Briggs Institute (JBI) checklist for case–control and cohort studies. Green indicates low risk, yellow indicates unclear risk, and red indicates high risk of bias for each domain. Yusuf et al., 2018 [21]; Weir et al., 2013 (USA) [34]; Lin et al., 2016 (China) [30]; Kulecka. et al., 2024 (Poland) [35]; Yang et al., 2023 (China) [37]; Mehta et al., 2024 (India) [22]; Sze et al., 2019 (USA) [5]; Nannini et al., 2021 (Italy) [31]; Song et al., 2018 (South Korea) [23]; Kim et al., 2020 (South Korea) [36]; Ohigashi et al., 2013 (Japan) [24]; Wang et al., 2025 (Chi-na) [32]; Du et al., 2022 (Chi-na) [33].

**Figure 2:**
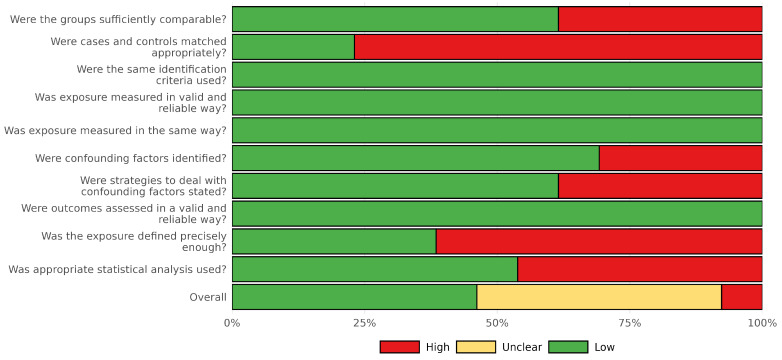
Risk-of-bias summary plot. Aggregate visualization of the proportion of studies rated as low, unclear, or high risk of bias for each JBI domain. The overall assessment reflects the distribution of risk levels across all included studies.

**Study Quality Assessment:** We evaluated the quality of studies using the Joanna Briggs Institute (JBI) Critical Appraisal Checklists for case–control and cross-sectional studies [29]. We assessed the study risk of bias using ten domains including selection of participants, measurement of exposure and outcome, identification and control of confounding variables, and appropriateness of statistical analysis. Each domain was rated as ‘high risk of bias’, ‘no risk of bias’ or ‘not applicable’. An overall risk of bias classification (low, unclear, high) was assigned based on the number and criticality of ‘no risk of bias’ ratings. Of the 13 studies, six were judged to be at low risk of bias [21,24,30,31,32,33], six at unclear risk [22,23,34,35,36], and one at high risk [37]. The most frequent sources of potential bias were related to matching of cases and controls, identification and control of confounding factors, and inadequate definition or duration of exposure. Assessments were performed independently by two reviewers, and discrepancies were resolved through discussion and consensus. We excluded all studies classified as low quality from the meta-analysis; however, all 13 studies were retained in the systematic review after careful re-evaluation of the single study assessed as having a high risk of bias. The distribution of judgments across studies and domains is presented in the traffic light plot (Figure 1) and summary plot (Figure 2) [38].

**Statistical Analysis:** This meta-analysis synthesized data from four observational studies [21,22,24,32] that measured fecal concentrations of acetate, propionate, and butyrate in patients with colorectal cancer compared with healthy controls. Altogether, the studies included 141 CRC cases and 98 controls, with sample sizes per group ranging from 14 to 93. Because the studies reported short-chain fatty acid levels in different units (µg/mL or µmol/g feces), the primary analysis was based on standardized mean differences (SMD) to enable direct comparison across studies.

All statistical analyses were conducted in R (version 4.4.1) using the metafor package. For each SCFA, a random-effects model with restricted maximum-likelihood (REML) estimation was applied to pool the SMD. The pooled SMD represents the average difference in metabolite levels between CRC and control groups, with negative values indicating lower levels in CRC. The random-effects approach accounts for between-study variability and provides a more conservative, generalizable estimate than a fixed-effects model. Ninety-five percent confidence intervals (95% CI) and *p*-values were calculated to assess the statistical significance of the pooled effects.

Heterogeneity across studies was evaluated using the estimated between-study variance (τ^2^), the proportion of total variability attributable to heterogeneity (I^2^), the ratio of total to within-study variance (H^2^), and Cochran’s Q statistic with corresponding degrees of freedom and *p*-value to test the null hypothesis of homogeneity. Study-level comparisons between CRC and control groups were assessed using Welch’s t-tests. Sensitivity analyses were performed with a leave-one-out procedure, recalculating the pooled estimate after sequential exclusion of each study.

As a secondary analysis, mean-difference (MD) models were fitted for the subset of three studies that reported results in the same unit (µmol/g feces; excluding [21] which used µg/mL). These analyses, also conducted under a random-effects framework, provide raw-scale estimates of group differences but are limited to directly comparable measurements. Forest plots were generated to visualize study-specific and pooled estimates for both SMD and MD analyses.

## 3. Results

**Figure 3:**
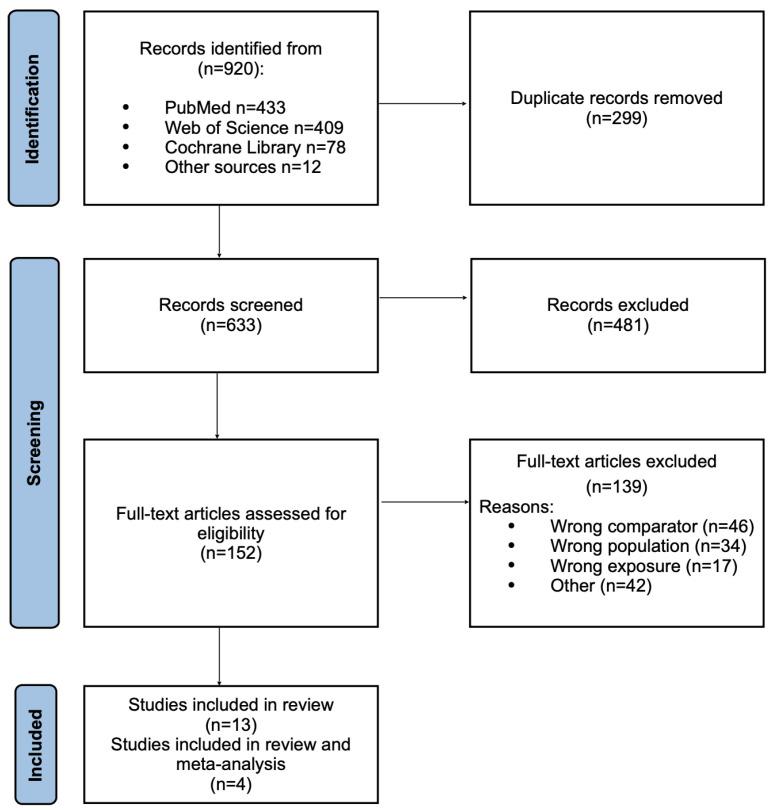
Preferred Reporting Items for Systematic Reviews and Meta-Analyses (PRISMA) flow diagram showing study identification, screening, eligibility assessment, and inclusion process.

**Study Inclusion and Characteristics of Included Studies:** A total of 920 records were identified through database searches, including PubMed (n = 433), Web of Science (n = 409), and the Cochrane Library (n = 78), as well as other sources (n = 12). After removal of 299 duplicates, 633 unique records remained for title and abstract screening. Of these, 481 were excluded for not meeting the inclusion criteria. The full texts of 152 articles were then assessed for eligibility, and 139 were excluded for the following reasons: wrong comparator (n = 46), wrong population (n = 34), wrong exposure (n = 17), or other reasons (n = 42). Ultimately, 13 [21,22,23,24,30,31,32,33,34,35,36,37] studies met the inclusion criteria for qualitative synthesis, and 4 [21,22,24,32] of these provided sufficient quantitative data for inclusion in the meta-analysis. Figure 3 presents PRISMA flowchart of study identification, screening, eligibility assessment, and inclusion process. The characteristics of the all 13 included studies are summarized in Table 1. Across these studies, sample sizes in the CRC groups ranged from 10 to 120 patients, while control groups ranged from 11 to 370 individuals. Some studies matched participants for age and sex, but this was not consistent across all datasets. In most cases, the CRC and control groups were of unequal size. Although all studies were described by their authors as case–control, they were methodologically cross-sectional comparative analyses, as fecal samples were collected after disease onset and no pre-diagnostic exposure assessment was performed. Of the included studies, 10 were conducted in Asia, 2 in Europe, and 1 in North America. Tumor staging was reported using diverse systems, including CRC stage according to AJCC Cancer Staging Manual, however most of the authors did not mention the edition they used [39], Duke’s classification [40], or the T characteristic of the AJCC colorectal cancer TNM system. Three studies did not report tumor stage at all. Similarly, tumor localization was classified heterogeneously—by right vs. left colon, colon vs. rectum, or proximal vs. distal colon—with variability across studies. In five studies, tumor location was not reported. The studies also differed in their exclusion criteria. Most excluded participants who had recently used antibiotics, typically within 1–3 months prior to stool collection. Several excluded patients who had undergone recent surgery, though the indications for exclusion varied. Some studies excluded only those previously operated for colorectal cancer, while others also excluded individuals operated for unrelated conditions. Comorbidity exclusions were inconsistent across studies: some removed participants with inflammatory bowel disease, metabolic disorders, or genetic cancer syndromes (e.g., FAP, Lynch syndrome), while others did not specify. Dietary restrictions were rarely controlled; when mentioned, exclusions most often applied to probiotic or yogurt consumption. A few studies excluded participants with recent chemo- or radiotherapy, but definitions of “recent” were variable. Only one study explicitly excluded participants based on smoking or alcohol consumption. In all included studies, stool samples were analyzed. Sampling was performed before surgery, bowel preparation, antibiotic exposure, or oncological treatment, ensuring that fecal SCFA levels reflected the untreated metabolic and microbial environment. The characteristics of the 13 included studies are summarized in Table 1.

The most frequently analyzed short-chain fatty acids were acetate, propionate, and butyrate, while several studies also reported total SCFAs or fecal pH. Analytical methods included validated techniques such as gas chromatography (GC), gas chromatography–mass spectrometry (GC–MS), gas chromatography–time-of-flight mass spectrometry (GC–TOF-MS), high-performance liquid chromatography (HPLC), ultra-performance liquid chromatography–mass spectrometry (UPLC–MS), and proton nuclear magnetic resonance spectroscopy (^1^H-NMR).

## 4. Systematic Review

**Acetic Acid:** All thirteen included studies assessed fecal acetic acid either quantitatively or descriptively, although only six provided exact numerical data. Units, measurement techniques, and analytical sensitivity varied substantially across studies, which limits direct comparability. Overall, the evidence regarding acetate alterations in CRC suggests significantly lower values in colorectal cancer patients compared to healthy control groups. Yang et al. found no significant differences in acetate between CRC and healthy controls, nor between diabetic and non-diabetic CRC subgroups (*p* > 0.05) [37]. Mehta et al. also reported no statistically significant difference in acetate concentrations between groups (*p* > 0.05) [22]. Sze et al., comparing both adenoma and carcinoma cohorts to healthy subjects, observed comparable acetate levels across all groups and found no significant difference in before and after treatment comparison of adenoma and cancer groups (*p* > 0.05) [5]. Song et al., similarly, detected no sex-dependent differences in fecal acetate concentrations (*p* > 0.05) [23]. Wang et al. did not report on significantly different acetate levels among patients with stage IV colorectal cancer in comparison to healthy control group (*p* > 0.05) [32].

By contrast, Ohigashi et al. demonstrated a significant reduction in acetate in CRC patients (49.3 ± 27.2 µmol/g) compared with healthy controls (59.6 ± 19.2 µmol/g; *p* = 0.002) [24]. Lin et al., using proton nuclear magnetic resonance spectroscopy, likewise found a significantly lower relative abundance of acetate in CRC (24 ± 6 a.u.) than in controls (45 ± 11 a.u., *p* < 0.05) [30]. Yusuf et al. reported lower, significant (*p* < 0.05), acetate concentrations in CRC patients (8.55 ± 3.06 µg/mL) compared with healthy controls (11.78 ± 4.61 µg/mL) [21]. Kulecka et al. observed a trend toward higher acetate levels in CRC samples, though no absolute concentrations or statistical comparisons were provided [35]. Weir et al. also observed a statistically significant increase in the relative proportion of acetate in CRC, though exact concentrations were not reported (*p* < 0.05) [34].

**Propionic Acid:** All included studies assessed fecal propionic acid levels or proportions in colorectal cancer patients compared with healthy controls, although not all reported exact concentrations. Units, measurement techniques, and analytical sensitivity varied substantially across studies, which limits direct comparability. Overall, the evidence regarding propionate alterations in CRC is inconsistent. Mehta et al. found similar propionate levels between CRC and control groups (*p* > 0.05) [22], and Song et al. likewise demonstrated no differences between sex groups in healthy and cancer groups, in propionate relative abundance (*p* > 0.05) [23]. Wang et al. reported similar propionate values in stool propionic acid without statistically significant difference between stage IV CRC patients and healthy control group (*p* > 0.05) [32]. Sze et al., comparing both adenoma and carcinoma cohorts to healthy subjects, observed comparable propionate levels across all groups and found no significant difference in before and after treatment comparison of adenoma and cancer groups (*p* > 0.05) [5]. Weir et al. also did not observe a statistically significant difference between the relative proportion of propionate in CRC, though exact concentrations were not reported (*p* > 0.05) [34].

In contrast, Ohigashi et al. provided the most detailed analysis of propionate in relation to clinical variables. They reported significantly lower fecal propionic acid concentrations in CRC patients (12.7 ± 8.1 µmol/g stool) than in healthy controls (19.8 ± 6.6 µmol/g; *p* < 0.001). No significant differences, however, were observed when comparing propionate levels across Duke’s stages or tumor locations (*p* > 0.05) [24]. Yusuf et al. reported a lower absolute concentration of propionic acid in CRC (5.61 ± 1.95 µg/mL) than in healthy controls (8.61 ± 3.40 µg/mL), with statistical significance (*p* = 0.008) [21]. Lin et al., using a method of ^1^H-NMR spectroscopy, also observed reduced concentrations of propionate in stage I/II CRC patient group (19 ± 6 a.u.) compared with controls (26 ± 7 a.u., *p* < 0.05) [30]. The remaining studies did not present quantitative propionate data but uniformly found no significant differences between groups. Yang et al. uniquely observed a significantly higher proportion of propionic acid in patients with both CRC and type 2 diabetes compared with those with CRC without concomitant diabetes (*p* < 0.05), the study did not find significant difference between CRC group and healthy subjects notwithstanding the diabetes status (*p* > 0.05), suggesting a metabolic rather than cancer-specific shift [37].

**Butyric Acid**: All included studies assessed fecal butyric acid levels or proportions in colorectal cancer patients compared with healthy controls, although not all reported exact concentrations. Units, measurement techniques, and analytical sensitivity varied substantially across studies, which limits direct comparability. Overall, the evidence regarding butyrate alterations in CRC suggests significantly lower values in colorectal cancer patients compared to healthy control groups. Yusuf et al. reported a significant reduction in fecal butyrate concentrations among CRC patients (3.79 ± 2.04 µg/mL) compared with healthy controls (6.81 ± 2.59 µg/mL; *p* = 0.002) [21]. Weir et al. also observed a statistically significant decrease in the relative proportion of butyrate in CRC, though exact concentrations were not reported (*p* < 0.05) [34]. Similarly, Lin et al., using proton nuclear magnetic resonance spectroscopy, found significantly lower relative abundance of butyrate in CRC (14 ± 6 a.u.) than in controls (23 ± 6 a.u., *p* < 0.05) [30]. Kim et al. observed a significant reduction in fecal butyrate in CRC patients, though exact concentrations were not provided (*p* < 0.05) [36]. Yang et al. demonstrated significantly lower butyrate levels in CRC patients and an even more pronounced decrease among those with concomitant type 2 diabetes (*p* < 0.05), suggesting that metabolic comorbidities further exacerbate butyrate depletion [37]. Nannini et al. reported decreased butyrate and isobutyrate in both adenoma and CRC groups compared with controls, though the reduction was greater in adenoma than in carcinoma, consistent with the concept of the “butyrate paradox” [31]—the phenomenon wherein proliferating cancer cells rapidly consume butyrate for glycolytic energy metabolism, despite its known anti-neoplastic effects in healthy colonocytes. This paradoxical relationship suggests that fecal butyrate depletion may reflect both impaired bacterial production and increased epithelial consumption by cancer tissue. In a larger and detailed biochemical analysis, Ohigashi et al. reported a markedly lower butyrate concentration in CRC (7.7 ± 4.7 µmol/g stool) than in controls (11.8 ± 4.9 µmol/g; *p* < 0.001), independent of Duke’s stage or tumor location [24].

Mehta et al. found no significant differences between groups, reporting comparable concentrations (CRC: 14.2 mmol/kg vs. controls: 13.5 mmol/kg, *p* > 0.05) [22]. Sze et al. detected no significant differences in butyrate concentrations among healthy controls, adenoma patients, and those with CRC, either before or after oncological treatment (*p* > 0.05) [5]. Song et al. similarly found no significant sex-related differences in fecal butyrate (*p* > 0.05) [23]. Wang et al. did not detect any significant differences in butyrate or other SCFAs between CRC and control groups (*p* > 0.05) [32].

In contrast, Kulecka et al. noted elevated isobutyrate and butyric acid levels in CRC samples, though without reporting absolute concentrations [35].

## 5. Meta-Analysis

A total of four observational studies [21,22,24,32] reporting fecal concentrations of acetate, propionate, and butyrate in patients with colorectal cancer and healthy controls were included in the quantitative synthesis. Combined, these studies represented 141 CRC cases and 98 controls. Because the studies used different measurement units (µg/mL or µmol/g feces), results were first analyzed using standardized mean differences (SMD) to allow comparison across datasets, followed by secondary analyses based on mean differences (MD) restricted to the subset of studies reporting values in µmol/g feces. Random-effects models were applied for all pooled estimates.

**Figure 4:**
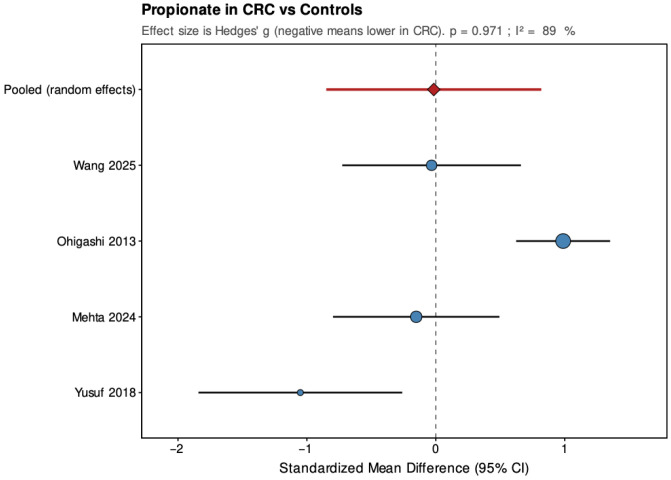
Forest plot summarizing pooled standardized mean difference (SMD) in propionate concentrations between patients with colorectal cancer and healthy controls. Figure presents SMD for acetate derived from random-effects models across four studies. Negative values indicate lower SCFA levels in CRC patients. CI, confidence interval; CRC, colorectal cancer. Blue circles indicate study-specific effect sizes, scaled by study weight; horizontal lines show 95% CIs. The red diamond denotes the pooled random-effects estimate with its 95% CI. Wang et al., 2025 [32]; Ohigashi et al., 2013 [24]; Mehta et al., 2024 [22]; Yusuf et al., 2018 [21].

**Figure 5:**
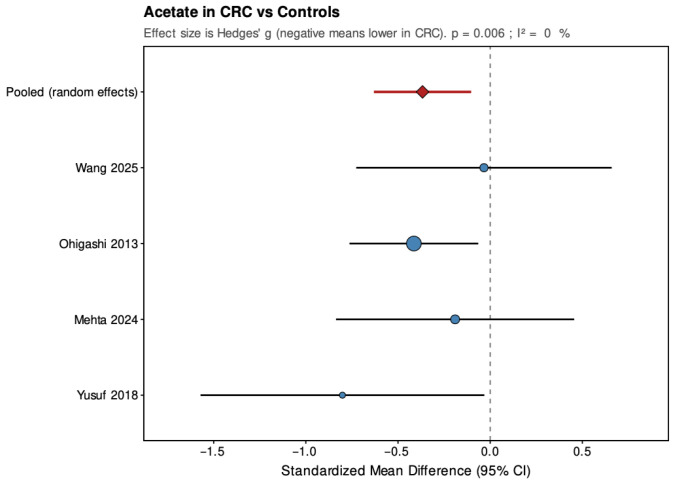
Forest plot summarizing pooled standardized mean difference (SMD) in acetate concentrations between patients with colorectal cancer and healthy controls. Figure presents SMD for acetate derived from random-effects models across four studies. Negative values indicate lower SCFA levels in CRC patients. CI, confidence interval; CRC, colorectal cancer. Blue circles indicate study-specific effect sizes, scaled by study weight; horizontal lines show 95% CIs. The red diamond denotes the pooled random-effects estimate with its 95% CI. Wang et al., 2025 [32]; Ohigashi et al., 2013 [24]; Mehta et al., 2024 [22]; Yusuf et al. [21].

**Figure 6:**
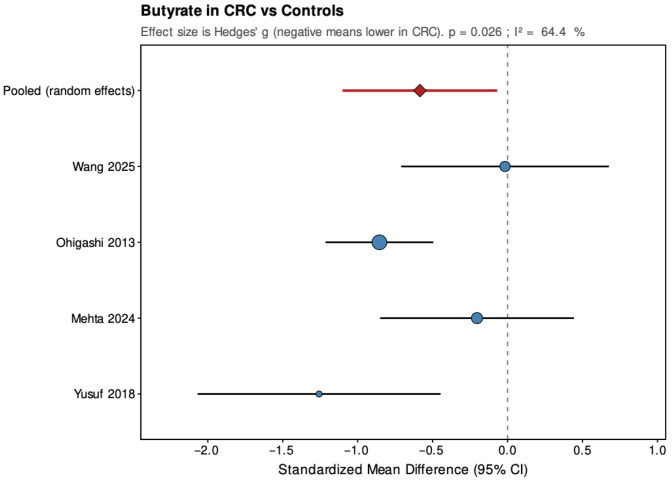
Forest plot summarizing pooled standardized mean difference (SMD) in butyrate concentrations between patients with colorectal cancer and healthy controls. Figure presents SMD for acetate derived from random-effects models across four studies. Negative values indicate lower SCFA levels in CRC patients. CI, confidence interval; CRC, colorectal cancer. Blue circles indicate study-specific effect sizes, scaled by study weight; horizontal lines show 95% CIs. The red diamond denotes the pooled random-effects estimate with its 95% CI. Wang et al., 2025 [32]; Ohigashi et al., 2013 [24]; Mehta et al., 2024 [22]; Yusuf et al. [21].

**Figure 7:**
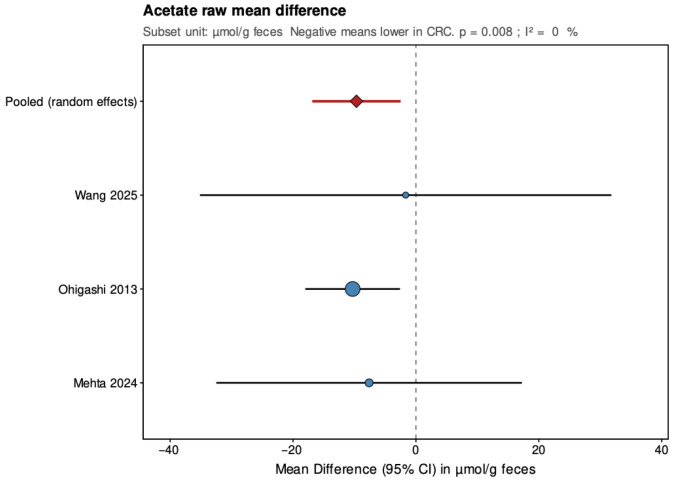
Forest plot summarizing pooled mean difference (MD) in propionate concentrations between patients with colorectal cancer and healthy controls. MD (µmol/g feces) for the subset of three studies reporting results in a common unit. Negative values indicate lower SCFA levels in CRC patients. CI, confidence interval; CRC, colorectal cancer. Blue circles indicate study-specific effect sizes, scaled by study weight; horizontal lines show 95% CIs. The red diamond denotes the pooled random-effects estimate with its 95% CI. Wang et al., 2025 [32]; Ohigashi et al., 2013 [24]; Mehta et al. 2024 [22].

**Figure 8:**
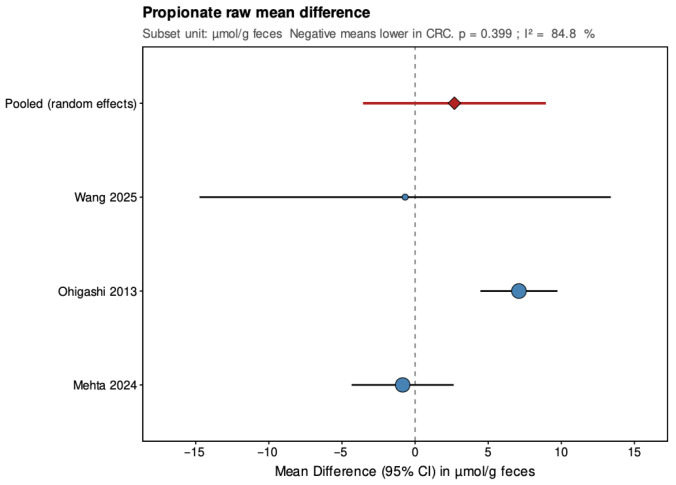
Forest plot summarizing pooled mean difference (MD) in acetate concentrations between patients with colorectal cancer and healthy controls. MD (µmol/g feces) for the subset of three studies reporting results in a common unit. Negative values indicate lower SCFA levels in CRC patients. CI, confidence interval; CRC, colorectal cancer. Blue circles indicate study-specific effect sizes, scaled by study weight; horizontal lines show 95% CIs. The red diamond denotes the pooled random-effects estimate with its 95% CI. Wang et al., 2025 [32]; Ohigashi et al., 2013 [24]; Mehta et al., 2024 [22].

**Figure 9:**
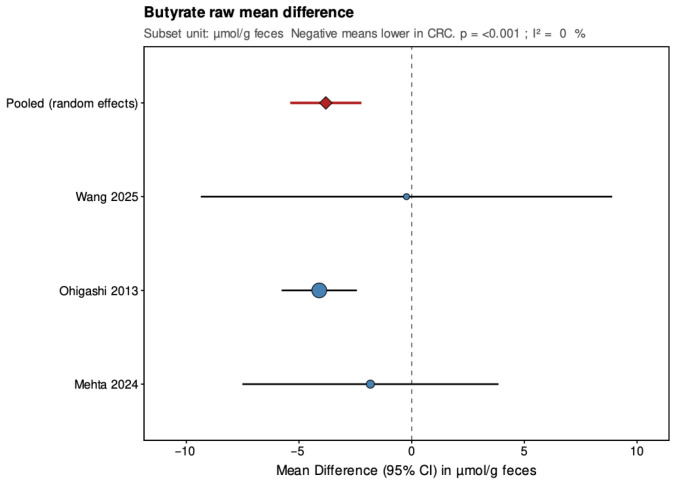
Forest plot summarizing pooled mean difference (MD) in butyrate concentrations between patients with colorectal cancer and healthy controls. MD (µmol/g feces) for the subset of three studies reporting results in a common unit. Negative values indicate lower SCFA levels in CRC patients. CI, confidence interval; CRC, colorectal cancer. Blue circles indicate study-specific effect sizes, scaled by study weight; horizontal lines show 95% CIs. The red diamond denotes the pooled random-effects estimate with its 95% CI. Wang et al., 2025 [32]; Ohigashi et al., 2013 [24]; Mehta et al., 2024 [22].

Pooled results from the random-effects models are summarized below (Figure 4, Figure 5, Figure 6, Figure 7, Figure 8 and Figure 9), with negative values indicating lower metabolite levels in CRC compared with healthy controls. The primary analyses focused on standardized mean differences (Hedges’ g, where approximately 0.2 is considered small, 0.5 moderate, and 0.8 large), while mean differences were additionally reported for the subset of studies that presented results in a common unit (µmol/g feces).

Across the four studies included, acetate showed a pooled SMD of −0.37 (95% CI: −0.63 to −0.10; *p* = 0.006), indicating a small but statistically significant reduction in CRC patients. Heterogeneity was low and non-significant (τ^2^ = 0, I^2^ = 0%, H^2^ = 0.823, Q = 2.47, df = 3, *p* = 0.481), suggesting consistent findings across studies. For propionate, the pooled SMD was −0.02 (95% CI: −0.85 to 0.82; *p* = 0.971), showing no significant overall difference between groups. The estimate was close to zero but imprecise, with high and statistically significant heterogeneity (τ^2^ = 0.62, I^2^ = 89%, H^2^ = 9.078, Q = 27.23, df = 3, *p* < 0.001), indicating substantial variation among studies. Butyrate demonstrated a pooled SMD of −0.59 (95% CI: −1.10 to −0.07; *p* = 0.026), representing a moderate, statistically significant reduction in CRC patients. Heterogeneity was moderate and statistically significant (τ^2^ = 0.177, I^2^ = 64.4%, H^2^ = 2.811, Q = 8.43, df = 3, *p* = 0.038), with all studies showing lower levels in CRC but varying in effect size.

In the secondary analyses limited to three studies reporting data in µmol/g feces, acetate showed a pooled MD of −9.68 (95% CI: −16.87 to −2.48; *p* = 0.008), again indicating a statistically significant reduction in CRC patients with low and non-significant heterogeneity (τ^2^ = 0, I^2^ = 0%, Q = 0.27, *p* = 0.873). Propionate had a pooled MD of 2.69 (95% CI: −3.56 to 8.94; *p* = 0.399), showing no significant difference and high heterogeneity (τ^2^ = 21.208, I^2^ = 84.8%, Q = 13.15, *p* = 0.001). Butyrate presented a pooled MD of −3.81 (95% CI: −5.39 to −2.23; *p* < 0.001), confirming a statistically significant reduction in CRC patients with low and non-significant heterogeneity (τ^2^ = 0, I^2^ = 0%, Q = 1.17, *p* = 0.556). Leave-one-out sensitivity analyses demonstrated that pooled SMD estimates for acetate and butyrate were robust, remaining consistent and statistically significant when any single study was removed. For propionate, the high heterogeneity made the pooled estimate more sensitive to individual studies. The MD results were directionally consistent with the SMDs and provided absolute differences for studies sharing comparable measurement units.

Overall, pooled analyses demonstrated that acetate and butyrate concentrations were significantly lower in CRC patients compared with healthy controls, corresponding to small and moderate effect sizes, respectively. Propionate showed no consistent pattern, with pooled estimates crossing the null line and substantial between-study heterogeneity. These findings indicate a reproducible reduction in acetate and butyrate across studies, while results for propionate remain inconclusive.

## 6. Discussion

Across the thirteen studies included in this review, fecal short-chain fatty acid profiles in colorectal cancer compared to healthy individuals show no uniform pattern, but several reproducible trends emerge. The meta-analysis of four studies demonstrated significantly lower (*p* < 0.05) fecal acetate and butyrate concentrations in CRC patients, while propionate showed no consistent difference. These findings indicate that global SCFA depletion, particularly of butyrate, accompanies colorectal carcinogenesis, though results remain heterogeneous across individual datasets. Butyrate, the most biologically active SCFA in the colon, serving as the primary energy substrate for colonocytes and exerts anti-inflammatory, anti-neoplastic, and barrier-protective effects [41,42], consistently appeared reduced in CRC patients in both the narrative synthesis and the meta-analysis (SMD = −0.59, *p* = 0.026). This observation aligns with its established role as a key energy source for colonocytes and as an epigenetic regulator that suppresses tumor growth through histone deacetylase inhibition [15]. The pattern supports the concept that reduced butyrate production or utilization reflects both the loss of butyrate-producing bacteria and impaired fermentation pathways in the tumor microenvironment [43,44]. In line with the ‘butyrate paradox,’ the same molecule that supports differentiation and apoptosis in healthy colonocytes may accumulate in glycolysis-dependent cancer cells, inducing growth arrest [45]. Thus, the fecal depletion observed here likely represents diminished microbial production rather than increased epithelial consumption.

Acetate, though the most abundant SCFA, showed a smaller and more variable reduction (SMD = −0.37, *p* = 0.006). Its high systemic turnover and diverse bacterial origin make fecal acetate more sensitive to dietary and metabolic variation [46,47]. Several included studies reported unchanged levels, suggesting that global fermentation capacity might persist despite microbial compositional shifts. Heterogeneity in dietary fiber intake may contribute to these discrepancies.

Propionate results were inconsistent (SMD ≈ 0, *p* = 0.97), with substantial heterogeneity across studies (I^2^ ≈ 89%). Propionate metabolism is primarily hepatic, serving as a gluconeogenic substrate, and fecal concentrations may not accurately reflect its overall production [46,47]. Some evidence suggests that metabolic comorbidities, such as diabetes, influence propionate levels more than cancer itself [37]. Hence, propionate alterations appear nonspecific for malignant transformation.

Collectively, this article and meta-analysis confirms that CRC is associated with lower fecal butyrate and, to a lesser extent, acetate, while propionate remains variable. These trends align with prior literature linking low SCFA output to dysbiosis and epithelial dysfunction in CRC [6,11,43].

This review is, to our knowledge, the first to quantitatively synthesize SCFA data comparing colorectal cancer patients to healthy subjects. The inclusion of only histologically confirmed CRC and healthy adult controls with additional colorectal adenoma group increases comparability. Several limitations constrain interpretation. Substantial heterogeneity was present across studies regarding sample collection, storage, analytical techniques, and reporting units, preventing absolute concentration pooling and inflating between-study variability. All included studies were cross-sectional or case–control, precluding causal inference. Dietary intake, antibiotic exposure, and probiotic use were inconsistently reported, introducing a high risk of residual confounding. Tumor stage, site, and treatment history were insufficiently standardized, limiting biological interpretability. Critically, the meta-analysis was based on only four independent datasets. Although sensitivity analyses indicated directional stability, the limited number of studies markedly reduces statistical power, precision, and external validity, and increases susceptibility to small-study effects. This is particularly relevant for propionate, where the absence of a significant signal should be interpreted with caution rather than as evidence of true biological neutrality. Accordingly, the quantitative findings should be regarded as hypothesis-generating rather than definitive. At the same time, this analysis represents the highest level of quantitative evidence that can currently be extracted from the available literature, and underscores the urgent need for larger, methodologically standardized studies in this field.

Our findings are consistent with earlier reviews. Previous work from Alvandi et al. [6] identified consistent decreases in the combined fecal concentration of acetate, propionate, and butyrate in CRC patients compared with healthy controls, and similar reductions among individuals at increased CRC risk. In contrast, our analysis added to existing knowledge by examining SCFAs separately rather than pooled total SCFA values. This approach allowed a more detailed assessment of metabolite-specific alterations and revealed that reductions are not uniform across all SCFAs. In particular, butyrate levels showed the most pronounced and consistent decrease in CRC, while acetate exhibited greater heterogeneity, it was still significantly decreased in CRC patients. Propionate results, however, were not sufficiently consistent, robust, or statistically significant to draw definitive conclusions. By focusing on single-metabolite patterns, our study adds evidence for the depletion of butyrate, and to lesser extent, acetate as a hallmark of CRC-associated dysbiosis Further progress requires methodological harmonization. Future studies should standardize stool collection timing, storage, and quantification protocols. Dietary data should be captured with validated food-frequency questionnaires, and antibiotic or probiotic use should be clearly excluded or adjusted for. Integrating SCFA quantification with microbiome sequencing would clarify microbial sources of each metabolite and strengthen causal inference. Longitudinal cohort designs, following individuals from a healthy state through adenoma to carcinoma, are essential to establish temporal relationships. Additionally, studies should explore tumor stage and tumor site specific patterns and evaluate whether fecal SCFAs recover after surgical or chemotherapeutic treatment. Although routine SCFA measurement is not yet clinically warranted, the observed depletion of fecal butyrate remains a biologically plausible and methodologically attractive lead in the search for non-invasive biomarkers of colorectal cancer. As a systematic review and meta-analysis, this work synthesizes the current state of evidence and extends it by providing quantitative estimates of SCFA alterations in CRC. Nevertheless, the available studies are heterogeneous in design, analytical methods, and reporting quality. Therefore, these results should be interpreted as hypothesis-generating rather than confirmatory. They highlight consistent patterns that warrant further mechanistic and translational exploration, rather than immediate clinical application. Future investigations should prioritize harmonized sampling protocols, standardized quantification techniques, and adequately powered, longitudinal designs to address the substantial methodological gaps identified. Beyond biomarker development, the findings reinforce the biological plausibility of preventive strategies targeting dietary fiber intake and restoration of butyrate-producing taxa, both of which are central to maintaining intestinal homeostasis. Once standardized, SCFA quantification may provide a valuable adjunct for understanding host–microbiome metabolic interactions in CRC pathogenesis, but robust validation remains essential.

**Conclusion:** Fecal SCFA levels, particularly butyrate and acetate, are consistently reduced in colorectal cancer, supporting the concept of impaired microbial fermentation in tumor-associated dysbiosis. Propionate alterations, nevertheless, appear nonspecific. Meta-analytic synthesis demonstrates a significant pooled decrease in two out three main SCFA concentrations among CRC patients compared with healthy controls, yet the methodological heterogeneity, small sample sizes, and incomplete reporting across primary studies constrain interpretability. These limitations underscore the need for standardized methodology to allow drawing more conclusions. Collectively, the evidence supports a mechanistic role of disrupted microbial fermentation in colorectal carcinogenesis and provides a conceptual foundation for biomarker research. However, future harmonized, longitudinal studies integrating dietary, microbiome, and metabolomic data are required before fecal SCFA profiling can be considered a reliable diagnostic or prognostic tool in clinical oncology.

## Figures and Tables

**Table 1:** Presents the characteristics of the all 13 studies that were analyzed in the systematic review, 4 of which were subsequently included into meta-analysis: study design, participant demographics, tumor features, key exclusion criteria, analytical methods. Abbreviations: CRC—colorectal cancer; CRA—colorectal adenoma; DM—diabetes mellitus; GI—gastrointestinal; NSAID—nonsteroidal anti-inflammatory drug; AJCC—American Joint Committee on Cancer; TNM—tumor–node–metastasis; FAP—familial adenomatous polyposis; GC—gas chromatography; GC–MS—gas chromatography–mass spectrometry; GC–TOF-MS—gas chromatography–time-of-flight mass spectrometry; HPLC—high-performance liquid chromatography; UPLC–MS—ultra-performance liquid chromatography–mass spectrometry; ^1^H-NMR—proton nuclear magnetic resonance.

Study and Location	Study Design	CRC Group Size (n)	Control Sample Size (n)	Mean Age (Years)	Sex (% of Man)	CRC Stage (as Reported)	Tumor Location (as Reported)	Key Exclusion Criteria (as Reported)	Measurement Method	Included in Meta-Analysis
Yusuf et al., 2018 (Indonesia) [21]	Case–control	14	14	CRC: 53.8 Healthy: 50.0	CRC: 72% Healthy: 64%	Not reported	Descending colon: 3 Rectum: 11	Antibiotic use within 1 month prior to sampling Yogurt or probiotic intake or laxative use within 5 weeks prior to sampling Chemotherapy or radiotherapy within the past 6 months	GC	Yes
Weir et al., 2013 (USA) [34]	Case–control	10	11	CRC: 63.7 Healthy: 40.7	CRC: 80% Healthy: 27.3%	TNM-T Tis: 1; T1: 2; T2: 3; T3: 4; T4: 2	Ascending colon: 3 Sigmoid colon: 4 Rectum: 3	Antibiotic use within 2 months prior to participation. Regular use of NSAIDs, statins, or probiotics Chronic bowel disorders, food allergies, or dietary restrictions Chemotherapy or radiotherapy before surgery	GC-MS	No
Lin et al., 2016 (China) [30]	Case–control	68	32	CRC: 51.3 Healthy: 50.6	CRC: 52.9% Healthy: 46.9%	AJCC I/II: 20 III: 25 IV: 23	Not reported	Use of antibiotics, NSAIDs, statins, or probiotics within 2 months before study participation Chemotherapy or radiotherapy prior to surgery	^1^H NMR	No
Kulecka et al., 2024 (Poland) [35]	Case–control (multi-arm)	40	40	CRC: 66.5 Healthy: 64.5 (medians)	CRC: 50% Healthy: 50%	Not reported	Not reported	Antibiotic use within 2 months prior to fecal sampling Inflammatory bowel disease or previous history of cancer	GC-MS	No
Yang et al., 2023 (China) [37]	Case–control (three-arm)	24	12	non-DM-CRC: 58.1 DM-CRC: 60.7 Healthy: 57.7	CRC: 50% DM+CRC: 50% Healthy: 50%	AJCC non-DM-CRC: I: 2; II: 4; III: 5; IV: 1 DM-CRC: I: 2; II: 2; III: 7; IV: 1	DM-CRC: Right-sided: 4 Left-sided: 8 non-DM-CRC: Right-sided: 3 Left-sided: 9	IBD, FAP or Lynch syndrome Antibiotic use within 2 weeks prior to sampling Severe cardiovascular disease Smoking or alcohol consumption	GC-MS	No
Mehta et al., 2024 (India) [22]	Case–control	18	19	CRC: 55.78 Healthy: 54.21	CRC: 55.6% Healthy: 68.4%	AJCC I: 1; II: 7; III: 2; IV: 8	Colon: 11 Rectosigmoid: 2 Rectum: 5	Multiple malignancies Family history of CRC (first-/second-degree relatives) IBD, chronic kidney or liver disease	GC-MS	Yes
Sze et al., 2019 (USA) [5]	Case–control + longitudinal subset	120	Healthy: 172 CRA: 198	Not reported	Not reported	Not reported	Not reported	Not reported	HPLC	No
Nannini et al., 2021 (Italy) [31]	Case–control (three-arm)	32	Healthy: 38 CRA: 16	CRC: 72 CRA: 59 Control: 47 (medians)	CRC: 68.8% CRA: 56.3% Control: 65.8%	TNM-T T0: 2; T1: 6; T2: 10; T3: 6; T4: 8	Not reported	Antibiotic, probiotic, or prebiotic use within the last 3 months prior to sampling IBD Other malignancies	^1^H NMR	No
Song et al., 2018 (South Korea) [23]	Case–control	26	Healthy: 28 CRA: 28	CRC: 59.7 CRA: 53.6 Healthy: 51.1	CRC: 61.5% CRA: 92.6% Healthy: 78.6%	AJCC I: 3; IIa: 5; IIc: 1; IIIb: 11; IIIc: 3; IVa: 3	Proximal colon: 3 Distal colon: 23	Antibiotic or probiotic use within the last 3 months Chronic gastrointestinal disorders Prior chemotherapy or radiotherapy	GC-MS	No
Kim et al., 2020 (South Korea) [36]	Case–control	32	40	CRC: 63.6 Healthy: 63.3	CRC: 62.5% Healthy: 55%	AJCC 0: 1 I: 7 II: 12 III: 9 IV: 3	Cecum: 2 Ascending colon: 6 Transverse colon: 1 Sigmoid colon: 12 Rectum: 7	Recurrent CRC post-surgery Prior chemotherapy Antibiotic use within 1 month prior to sampling GI disease	GC–TOF-MS	No
Ohigashi et al., 2013 (Japan) [24]	Case–control	93	Healthy: 22 CRA: 27	CRC: 68.9 CRA: 66.6 Healthy: 65.6	CRC: 52.7% CRA: 50% Healthy: 59.3%	Dukes A: 36; B: 19; C: 24; D: 14	Cecum: 5 Ascending colon: 21 Transverse colon: 9 Descending colon: 3 Sigmoid colon: 20 Rectum: 35	History of colectomy or proctectomy Obstructive CRC Antibiotic treatment during hospitalization	HPLC	Yes
Wang et al., 2025 (China) [32]	Case–control	16	16	CRC: 64.2 Healthy: 62.7	CRC: 56.3% Healthy: 62.5%	AJCC IV: 16	Not reported	Antibiotic or probiotic use within 3 months prior to sampling Metabolic disease Other malignancy	GC-MS	Yes
Du et al., 2022 (China) [33]	Case–control	30	33	CRC: 56 Healthy: 59	CRC: 40% Healthy: 39%	AJCC I: 2; II: 12; III: 14; IV: 2	Right-sided: 5 Left-sided: 25	Immunodeficiency, cardiovascular disease, DM, or hypertension Other malignancy Antibiotic use within 2 months prior to sampling Regular use of NSAIDs, probiotics, or statins Preoperative chemotherapy or radiotherapy	UPLC–MS	No

## Data Availability

The raw data supporting the conclusions of this article will be made available by the authors on request.

## Supplementary Materials

The following supporting information can be downloaded at https://www.mdpi.com/article/10.3390/jcm14248949/s1, Table S1: PRISMA 2020 checklist.
